# Gender and ethnoracial disparities in Veterans’ trauma exposure prevalence across differing life phases

**DOI:** 10.1186/s40621-025-00561-5

**Published:** 2025-02-03

**Authors:** Fernanda S. Rossi, Yael I. Nillni, Alexandria N. Miller, Annie B. Fox, Johanne Eliacin, Paula P. Schnurr, Christopher C. Duke, Jaimie L. Gradus, Tara E. Galovski

**Affiliations:** 1 Department of Psychiatry and Behavioral Sciences, Stanford University School of Medicine, Stanford, CA USA; 2 Center for Innovation to Implementation, VA Palo Alto Health Care System, Menlo Park, CA USA; 3 Women’s Health Sciences Division, National Center for PTSD, VA Boston Health Care System, Boston, MA USA; 4 Department of Psychiatry, Boston University Chobanian and Avedisian School of Medicine, Boston, MA USA; 5 School of Healthcare Leadership, MGH Institute of Health Professions, Boston, MA USA; 6 Center for Health Information and Communication, Health Services Research & Development, Richard L Roudebush VA Medical Center, Indianapolis, IN USA; 7 Regenstrief Institute, Indianapolis, IN USA; 8 National Center for PTSD, White River Junction, VT, USA; 9 Geisel School of Medicine at Dartmouth, Department of Psychiatry, Hanover, NH USA; 10 Altarum, Ann Arbor, MI USA; 11 Boston University School of Public Health, Boston, MA USA

**Keywords:** Veterans, Trauma exposure, Gender, Race, Ethnicity, Life phases, Military service

## Abstract

**Background:**

Veterans show increased vulnerability to trauma exposure. Yet, there is limited research examining Veterans’ prevalence of experiencing different trauma exposure types by race, gender, and ethnicity and across unique phases of life (i.e., pre-military service, during service, and post-service). This study compares trauma exposure prevalence across women and men Veterans of differing ethnoracial identities (i.e., white, Black, Hispanic) within three life phases (i.e., pre-military service, during military service, post-military service).

**Methods:**

This study examined survey data from 3,544 Veterans (1,781 women; 1,686 men) across six discrete data collection points (between August 2018 to March 2022). Surveys were mailed nationally and oversampled for women (51.6%) and Veterans living in high crime areas (67.6%). Veterans reported on their exposure to various trauma types (e.g., sexual assault, physical assault, community violence, captivity, serious accident, witnessing violent death) at each wave of data collection using items from a modified Life Events Checklist. Veterans also reported on demographic information (e.g., gender, race, ethnicity). Chi-square analyses were conducted to compare prevalence of reported exposure to each trauma type within each life phase across gender and ethnoracial groups.

**Results:**

There were significant differences in trauma exposure prevalence across: (1) men vs. women Veterans; (2) white vs. Black vs. Hispanic Veterans; (3) Black vs. Hispanic vs. white women Veterans; and (4) Black vs. Hispanic, vs. white men Veterans. For example, in this study, Black men Veterans reported higher prevalence of intimate partner physical assault exposure pre-service (14.8%) and post-service (27.1%) than White men Veterans (9.0% and 13.8%; prevalence ratios = 1.64, 95% CI = 1.17, 2.32 and 1.96, 95% CI = 1.53, 2.51). White women Veterans were less likely to witness a violent death pre-service (11.5%) than Black (21.1%; prevalence ratio = 1.83, 95% CI = 1.42, 2.37) or Hispanic (18.1%) women Veterans.

**Conclusions:**

Findings help uncover disparities within Veteran subgroups. They inform mental health treatment and prevention services to better meet the needs of all Veterans across differing life phases.

## Introduction

Nearly 70% of Americans experience a traumatic event at some point in their lifetime [1]. Some of the most frequently experienced traumatic events include witnessing someone being badly injured or killed, seeing a dead body or body parts, and childhood sexual abuse. Less frequently experienced traumatic events include being kidnapped or having prisoner of war status [2]. The prevalence of exposure to different types of traumatic events varies by a host of individual, demographic variables, such as gender, race, and ethnic backgrounds. Understanding prevalence of exposure to different traumatic events and how prevalence may differ within unique phases of life and across race, gender, and ethnicity is critical in uncovering potential disparities within subgroups of the population, particularly for those who are most vulnerable. This study seeks to describe differences in the prevalence of experiencing different types of traumatic events across gender, ethnic, and racial subgoups within a sample of Veterans. The military population is known for its high prevalence of exposure to trauma, and this will be the first study to capture differences in exposure across subgroups within unique phases of life. Information from this study will help elucidate potential patterns of trauma exposure in the Veteran population to help inform health-related resources and policies.

In the general population, consistent gender differences in exposure to trauma are observed. Men are overall more likely to experience traumatic events [2–4], and there are gender differences in the exposure to certain types of traumatic events. Compared to men, women have a higher prevalence of exposure to sexual violence perpetrated by an intimate partner, rape, sexual assault, childhood parental neglect, and childhood physical abuse [3–6]. In contrast, men, have a higher prevalence of exposure to physical assault, combat, being threatened with a weapon, being badly injured or killed, being involved in a fire, flood, or natural disaster, and being involved in a life-threatening accident [3–5].

Racial and ethnic differences are also evident in the prevalence of trauma exposure in the general population. A study using nationally representative data found that, compared to non-Hispanic Whites, Hispanic and non-Hispanic Black individuals are more likely to be exposed to child maltreatment and witness domestic violence as children [7]. In another study, Black individuals were more likely to be exposed to participation in organized violence (e.g. combat experience, witnessed atrocities) and sexual violence compared to White and Hispanic individuals [8]. Hispanic individuals were more likely to experience physical violence compared to White and Black individuals, and White individuals were most likely to experience accidents and injuries compared to Black and Hispanic individuals [8].

Veterans represent a specific subpopulation with increased vulnerability to trauma exposure [9]. In a nationally representative sample of Veterans, most (over 93%) had been exposed to at least one traumatic event in their lifetime, and many had experienced multiple events (average = 3.2 traumas) [10]. However, there is limited research understanding the prevalence of trauma exposure for Veterans across unique phases of life (i.e., pre-military service, during service, and post-service). Specifying prevalence of exposure to different types of traumas within the high-risk military population before, during, and after service is a critical step in prevention and intervention efforts.

An epidemiologic study examining retrospectively-reported trauma exposure among Veterans prior to entering service found that, compared to civilians, Veterans experienced higher rates of childhood sexual abuse, childhood physical abuse, and exposure to domestic violence [11]. Once in service, Veterans have an increased likelihood of exposure to traumas that are unique to military service, such as combat-related events and sexual trauma. Combat trauma is one of the most frequently experienced traumas, particularly by men, and is reported by 11.9% of Veterans [10, 12]. In a study of Veteran primary care patients, 53.3% of men and 13.2% of women reported combat trauma [12]. Military sexual trauma (MST), defined by The Department of Veterans Affairs (VA; U.S. Code 1720D) [13] as experiences of sexual assault or harassment that occur during military service, occurs far too frequently during service with substantially higher prevalence estimates for women than men. A recent population-based study found that approximately 7.5% of Veterans reported MST [14]. When considered separately, approximately 7.1% reported experiences of harassment (43.2% of women and 3.2% of men) and 2.7% experienced sexual assault (17% of women and 1.1% of men) [14]. After separation from military service, Veterans may also have increased exposure to certain trauma types, such as intimate partner violence (IPV). For example, in a study examining survey data from eight US states, nearly one-third of women Veterans reported lifetime sexual and physical IPV compared to less than one-quarter of civilian women [15]. Taken together, this body of work highlights Veterans’ differential heightened prevalence of exposure to specific trauma types within unique phases of life (pre-military service, during service, or post-military service), though, more work is needed directly comparing exposure to various trauma types across life phases.

There is also limited research comparing exposure to different trauma types across life phases by Veteran subgroups across race, gender, and ethnicity. Studies that did examine trauma exposure by Veteran subgroups across race, gender, and/or ethnicity do not specify life phases. For example, in a study that examined lifetime trauma exposure via phone interviews with 865 Veteran primary care patients from four VA medical centers, men reported more combat exposure, while women reported more childhood sexual abuse, adult sexual abuse, and physical assault with no weapon [12]. In this study, it is unclear when the sexual and physical assault occurred (e.g., pre-service, during service, post-service) for Veterans and if gender differences in exposures to sexual and physical assault persist over differing life phases. Indeed, it is possible that exposure to physical assault may be more heightened for women compared to men only during one specific life phase and that those differences disappear during other phases. Another study, that included the same sample and combined race and gender, found that Black women had higher rates of physical assault relative to White women [16]. Similar to the first study, this study raises questions about when the physical assault occurred (e.g., pre-service, during service, post-service) and whether Black women are more vulnerable to physical assault only during certain phases in the lifespan.

Understanding Veterans’ trauma exposure prevalence within life phases and across Veteran subgroups can shed light on whether prevalence estimates vary by race, gender, and ethnicity within life phases (e.g., pre-service, during service, post-service). Such information is critical in uncovering potential disparities within subgroups of the Veteran population. Such knowledge could enhance the tailoring of mental health treatment and prevention services to better meet the needs of all Veterans across their different life phases. For example, understanding disparities across Veteran subgroups could provide insight on how trauma-focused treatments may need to be adapted so that they are culturally-sensitive and responsive to diverse Veterans. It could inform which Veteran subgroups require more intensive trauma-focused prevention services and during which life phases. It could also inform the development of new policies that enhance access to trauma-focused treatments for Veteran subgroups in need.

This study sought to fill this gap by using the Longitudinal Investigation of Gender, Health, and Trauma (LIGHT) study, a national, longitudinal, mail-based survey of Veterans [17], to compare prevalence of trauma exposure across women and men Veterans of differing ethnoracial identities within three life phases across the lifespan (i.e., pre-military service, during military service, post-military service). Overall, this study aimed to: (1) compare prevalence of trauma exposure by gender within each life phase; (2) compare prevalence of trauma exposure by race/ethnicity within each life phase; and (3) compare prevalence of trauma exposure by race/ethnicity in each life phase among women and men separately.

This study is unique and further advances the literature in several important ways. First, we sampled for veterans across the full range of neighborhood contexts (e.g., rural, suburban neighborhoods) and oversampled for veterans living in high crime communities, who may be more likely to experience violent crimes. This allowed us to assess the prevalence of trauma exposure post-military service in these differing neighborhood contexts. Second, we oversampled for women Veterans, which provided us with a sufficiently robust sample to examine trauma exposure in women and men. Finally, we used an intersectional lens to compare trauma exposure in women and men Veterans of differing races and ethnicities. Our use of an intersectional lens in this study is based on the premise that human experience can be more adequately understood only when considering our multiple social positions (e.g., race, gender) [18]. We focused on gender and race/ethnicity given the known impact of these identities on social position [18]. This study adds critical information to the literature on trauma exposure among Veterans as few studies are able to examine the intersectionality of gender and race/ethnicity given small sample sizes.

## Methods

### Study sample

This prospective study asked Veterans to report on their trauma exposure history at six discrete data collection points (between August 2018 to March 2022) as part of the LIGHT survey study, an ongoing longitudinal study of Veterans. The LIGHT study aims to examine the impact of community violence exposure on Veterans’ mental health. Therefore, LIGHT oversampled for women and men Veterans living in high crime neighborhoods based on addresses/zip codes reflecting high and not high crime areas [17]. See Galovski et al. for additional recruitment and sampling details [17].

A national sample of 17,178 Veterans between the ages of 18 to 50 were invited to participate (60.4% high crime, 36.9% not high crime) using the Veterans Affairs/Department of Defense Identity Repository (VADIR), a VA-managed dataset of all separated service members, and 3,544 Veterans enrolled at Time 1 (21% response rate, which is consistent with response rates in other survey studies (20− 30%) [34]). Veterans were followed over time with 2,358 Veterans participating at Time 2, 1,924 at Time 3, 1,703 at Time 4, 1,553 at Time 5, and 1,485 at Time 6. The 3,544 Veterans (1,781 women; 1,686 men) who participated in at least the first (Time 1) wave of data collection were included in this analysis. Because sample sizes were too small to examine different gender identity categories, 57 Veterans who indicated non-matching responses on the sex and gender variables (e.g., male as gender and female as sex) or had missing data on these variables were excluded from analyses stratified by gender. Similarly, 440 Veterans who had either missing race/ethnicity data or selected a race category for which the sample size was too small to examine (i.e., Native American or Alaska Native, Asian, West Asian, Middle Eastern, or North African, Native Hawaiian, other Pacific Islander, and other) were excluded from analyses stratified by race/ethnicity. We compared Veterans who participated in Time 1 versus those who participated in at least one other wave of data collection (Times 2–6), as some Veterans were lost to follow-up, and found no significant differences between the two samples on race, gender, and age. This study was approved by the VA Boston Healthcare System Institutional Review Board.

### Measures

#### Demographic and military characteristics

Demographic and military information, including gender, race, ethnicity, age, education, income, branch of military service, military occupation, and deployment were self-reported at Time 1. For race, respondents were given a list of options to select from and could write-in a response if “other” was selected. As described above, we excluded race categories for which the sample size was too small to examine and focused only on White and Black respondents. Next, we combined the race and ethnicity variables to form a single variable representing three categories: non-Hispanic White alone (*n* = 1,919), non-Hispanic Black alone (*n* = 743), and Hispanic with any racial identity (*n* = 422), including Black and White respondents (hereafter referred to as White, Black, and Hispanic).

#### Trauma exposure

Participants were followed over time and at each wave of data collection, participants provided information about their exposure to trauma. At Times 2–6, participants were asked to report on their exposure to each trauma type since the last survey. This information was collapsed with Time 1 data. The following trauma types were examined: sexual assault by a non-intimate partner, sexual assault by an intimate partner, physical assault by a non-intimate partner, physical assault by an intimate partner, community violence, witnessed violent death, captivity, and serious accident. We focused on these trauma types due to their potential relevance to the Veteran population (e.g., Veterans are at high risk for interpersonal violence exposure) [10], because these traumas can occur during each life phase (e.g. combat would only occur during military service), and to expand the Veteran literature in areas that have received less attention (e.g., exposures to community violence) [17]. These trauma types were assessed using items from a modified and unique version of the Life Events Checklist (LEC-5) created for this study [19]. The LEC-5 is a self-report measure assessing traumatic event exposure across events known to potentially cause posttraumatic stress disorder or distress [19]. Our modified version included examples of each trauma type to help define the trauma type for participants (e.g., examples for captivity included being kidnapped, held hostage, prisoner of war). It included a new item assessing for exposure to community violence with the examples: terrorist attack, bombing, and riots. It also included two new items regarding sexual assault from an intimate partner and physical assault from an intimate partner. These two items were based on the United States’ Centers for Disease Control and Prevention’s definition of intimate partner violence experience [20] and adapted from the Lifetime Trauma Interview for Intimate Partner Violence Survivors [21].

Additionally, at Time 1, our modified version of the LEC-5 asked participants to indicate how many times they were exposed to each trauma type on a scale from 0 (*not at all*) to 3 (*many times*) across five time periods (i.e., childhood (i.e., under age 18), age 18 to enlistment, during military service, after military service, and in the last three months). These Time 1 data were collapsed to indicate trauma exposure to each trauma type across three life phases (i.e., *pre-military service*, which included childhood and age 18 to enlistment; *during military service*; and *post-military service*, which included after military service and in the last three months). For each of these three life phases, a dichotomous variable was created reflecting whether the participant had been exposed to the trauma at least once in the life phase (1 = response of 1-*once or twice*, 2-*several times*, or 3-*many times* recorded at any relevant time period) or had no exposure to that type of trauma in the life phase (0 = response of 0-*not at all*). In other words, Veterans who reported more than one exposure to a trauma type or the same traumatic experience multiple times across surveys within a life phase were coded as *1 = exposed*, regardless of exposure frequency. For example, a veteran reporting one serious accident during military service and a veteran reporting five serious accidents during military service were both coded as *1 = exposed* to a serious accident during military service. Trauma exposure data from Times 2–6 were collapsed with Time 1 data to inform post-military service trauma exposure. That is, Time 1 data informed trauma exposure in all three life phases while Times 2–6 data were used to further inform trauma exposure post-military service.

### Statistical analysis

We conducted 2 × 2 independent chi-squared analyses to compare prevalence of reported exposure to each trauma type within each timeframe across gender. We also conducted 3 × 2 independent chi-squared analyses to compare prevalence of reported exposure to each trauma type within each timeframe across ethnoracial groups. Additionally, we examined prevalence of reported exposure to trauma types across ethnoracial groups in each timeframe stratified by gender. Veterans with missing data on a particular trauma type were excluded from any corresponding analyses.

Follow-up analyses for significant chi-squared tests included a series of pairwise comparisons investigating differences in trauma exposure prevalence across groups. We applied a Bonferroni correction for each set of analyses (i.e., set 1: prevalence across gender; set 2: prevalence across race/ethnicity; set 3: prevalence across ethnoracial groups of women; set 4: prevalence across ethnoracial groups of men) to adjust for multiple comparisons using a critical alpha of *p* <.0167 for significance. We also calculated prevalence ratios (PR) based on prevalence of exposure for each trauma type and timeframe across groups as well 95% confidence intervals (CI) for each PR. PRs compare the prevalence of an outcome (e.g., trauma exposure) between groups. In PRs comparing gender, men were used as the referent group. In PRs comparing ethnoracial groups, White Veterans were used as the referent group.

## Results

### Demographic and military characteristics

Table 1 provides the demographic and military characteristics of the sample according to ethnoracial groups. Groups differed based on age, gender, education, income, branch in military service, military occupation, and deployment. However, all differences were small with Cramer’s V ranging from 0.058 to 0.314.

**Table 1:** Demographic Differences by Race/Ethnicity Group

Variables	**Black ** **(non-Hispanic)**		**Hispanic ** **(any race)**			**White** **(non-Hispanic)**			
	n=743		n=422			n=1919			
	n	%	n	%	v^1^	n	%	v^1^	v^2^
**Age**					.171***			.099***	.058*
18–34	223	30.76%	192	46.04%		779	41.09%		
34–49	477	65.79%	223	53.48%		1077	56.80%		
50+	25	3.45%	2	0.48%		40	2.11%		
**Gender**					.092**			0.117***	.027
Male	284	38.96%	202	48.44%		978	51.99%		
Female	445	61.04%	215	51.56%		903	48.01%		
**Branch**					.148***			.146***	.095***
Army	434	58.57%	222	52.73%		866	45.25%		
Marine Corps	40	5.40%	55	13.06%		181	9.46%		
Navy	138	18.62%	68	16.15%		342	17.87%		
Air Force	126	17.00%	70	16.63%		480	25.08%		
Coast Guard	3	0.40%	6	1.43%		45	2.35%		
**Mil Occupation**					.125***			.137***	.024
Combat arms	83	11.84%	75	18.66%		389	21.13%		
Combat support	224	31.95%	149	37.06%		673	36.56%		
Service support	394	56.21%	178	44.28%		779	42.31%		
**Deployed**	377	52.80%	237	58.81%	.058	1180	62.97%	.093***	.033
**Education**					.096**			.162***	.074**
High School	92	12.57%	32	7.77%		135	7.15%		
Some College	375	51.23%	198	48.06%		741	39.23%		
College +	265	36.20%	182	44.17%		1013	53.63%		
**Income**					.247***			.314***	.105***
Less than $24.9k	212	29.61%	81	19.90%		223	11.91%		
$25k - $54.9k	287	40.08%	108	26.54%		482	25.75%		
$55k - $99.9k	158	22.07%	130	31.94%		597	31.89%		
$100k+	59	8.24%	108	21.62%		570	30.45%		

*Note.* v1 = Cramer's V compared to Black; v2 = Cramer's V compared to Hispanic; not all counts sum to the total N due to missing data on individual survey questions.* *p* <.05** *p* <.01*** *p *< 001

### Differences in trauma exposure across gender

Table 2 shows trauma exposure prevalence, prevalence ratios, and 95% CIs for men and women by trauma type and life phase. Note that only statistically significant differences are described below. Non-significant differences are reported in the tables. Men, compared to women, were more likely to experience a serious accident and physical assault by someone other than an intimate partner as well as witness violent death in all three life phases (i.e., pre-service, during service, and post-service). For example, 53.3% of men reported a serious accident during service compared to 40.8% of women (PR = 0.77, 95% CI = 0.71, 0.82). Men (30.1%) were also more likely than women (21.4%) to experience community violence during service (PR = 0.71, 95% CI = 0.63, 0.80). On the contrary, women, compared to men, were more likely to experience physical assault by an intimate partner, sexual assault by an intimate partner, and sexual assault by someone other than an intimate partner in all three life phases. For example, 29.6% of women reported physical assault by an intimate partner during service compared to 14.5% of men (PR = 2.04, 95% CI = 1.78, 2.33). Additionally, women (3.5%) were more likely than men (2.0%) to be held captive prior to entering service (PR = 1.78, 95% CI = 1.17, 2.70).

**Table 2:** Trauma prevalence for veterans by trauma type, life phase, and gender

	Women (*N*=1781)						Men (*N*=1686)				
	%	Lower 95% CI	Upper 95% CI	Prev. Ratio	Lower 95% CI	Upper 95% CI	%	Lower 95% CI	Upper 95% CI	*p*	Cramer’s V
Serious Accident											
Pre-Service	47.3	45	49.7	0.87	0.82	0.93	54.4	52	56.8	**<0.001**	0.071
Service	40.8	38.5	43	0.77	0.71	0.82	53.3	50.9	55.6	**<0.001**	0.125
Post-Service	34.8	32.6	37	0.84	0.77	0.92	41.3	38.9	43.6	**<0.001**	0.067
Witnessed Violent Death											
Pre-Service	15.9	14.2	17.6	0.64	0.56	0.74	24.8	22.7	26.9	**<0.001**	0.11
Service	27.2	25.1	29.2	0.62	0.56	0.68	44.2	41.8	46.6	**<0.001**	0.178
Post-Service	22.5	20.6	24.5	0.81	0.72	0.91	27.7	25.6	29.8	**<0.001**	0.06
Community Violence											
Pre-Service	11.9	10.4	13.4	1.02	0.85	1.23	11.6	10.1	13.2	0.799	0.004
Service	21.4	19.5	23.4	0.71	0.63	0.80	30.1	27.9	32.3	**<0.001**	0.099
Post-Service	20.1	18.2	22	0.89	0.78	1.01	22.7	20.7	24.7	0.066	0.031
Captivity											
Pre-Service	3.5	2.6	4.3	1.78	1.17	2.70	2	1.3	2.6	**0.006**	0.047
Service	2.1	1.5	2.8	1.24	0.77	2.00	1.7	1.1	2.3	0.377	0.015
Post-Service	3.3	2.4	4.1	1.31	0.88	1.93	2.5	1.7	3.2	0.178	0.023
Sexual Assault (non-IP)											
Pre-Service	40.4	38.1	42.7	3.02	2.64	3.45	13.4	11.8	15	**<0.001**	0.303
Service	28.1	26	30.2	6.41	5.07	8.11	4.4	3.4	5.4	**<0.001**	0.319
Post-Service	9.1	7.8	10.4	2.89	2.14	3.92	3.1	2.3	4	**<0.001**	0.123
Physical Assault (non-IP)											
Pre-Service	20.7	18.8	22.6	0.72	0.64	0.82	28.6	26.4	30.7	**<0.001**	0.091
Service	12.5	10.9	14	0.50	0.43	0.58	25	23	27.1	**<0.001**	0.162
Post-Service	10.2	8.8	11.6	0.56	0.47	0.66	18.3	16.5	20.2	**<0.001**	0.116
Sexual Assault (IP)											
Pre-Service	18.7	16.9	20.5	4.93	3.80	6.38	3.8	2.9	4.7	**<0.001**	0.234
Service	20.5	18.6	22.4	6.64	5.01	8.82	3.1	2.3	3.9	**<0.001**	0.268
Post-Service	17.5	15.7	19.2	2.86	2.31	3.54	6.1	5	7.3	**<0.001**	0.175
Physical Assault (IP)											
Pre-Service	23.5	21.6	25.5	2.20	1.88	2.59	10.7	9.2	12.2	**<0.001**	0.17
Service	29.6	27.5	31.7	2.04	1.78	2.33	14.5	12.8	16.2	**<0.001**	0.181
Post-Service	25.2	23.2	27.2	1.41	1.24	1.61	17.9	16	19.7	**<0.001**	0.089

Note. Pairwise comparisons use a Bonferroni correction with a critical alpha of *p* <.0167; prevalence ratios for Female use Male as the reference category; IP = intimate partner

### **Differences in trauma exposure across ethnoracial groups**

Table 3 shows trauma exposure prevalence, prevalence ratios, and 95% CIs for White, Black, and Hispanic Veterans by trauma type and life phase. Prior to entering service, Black and Hispanic Veterans, compared to White Veterans, were more likely to experience physical assault from an intimate partner (21.5%, 19.4% vs. 14.9%; PRs = 1.45, 95% CI = 1.22, 1.73 and 1.31, 95% CI = 1.05, 1.63) and community violence (13.7%, 13.5% vs. 10.0%; PRs = 1.38, 95% CI = 1.10, 1.73 and 1.36, 95% CI = 1.03, 1.79) as well as witness violent death (26.1%, 22.7% vs. 16.2%; PRs = 1.62, 95% CI = 1.38, 1.89 and 1.41, 95% CI = 1.15, 1.73). Additionally, during the pre-service phase, Black Veterans were more likely than White Veterans to experience sexual assault from someone other than an intimate partner (31.5% vs. 24.3%; PR = 1.30, 95% CI = 1.14, 1.48). While in service, Black Veterans were more likely to experience physical assault (26.5%) by an intimate partner compared to White Veterans (20.0%; PR = 1.33, 95% CI = 1.14, 1.54). After leaving service, Black Veterans across the full range of neighborhood contexts (28.8%) were more likely than White Veterans (22.7%) to witness violent death (PR = 1.27, 95% CI = 1.10, 1.46). They were also more likely than White or Hispanic Veterans across the full range of neighborhood contexts, post-service, to experience a serious accident (44.7% vs. 37.4%, 34.5%; PR = 1.30, 95% CI = 1.17, 1.43) and physical assault by an intimate partner (31.8% vs. 21.1%, 16.8%; PR = 1.89, 95% CI = 1.64, 2.19) and to be held captive (4.8% vs. 2.1%, 1.9%; PR = 2.58, 95% CI = 1.64, 4.07). Hispanic Veterans across the full range of neighborhood contexts (21.1%) were more likely than White Veterans across the full range of neighborhood contexts (16.8%) to experience physical assault by an intimate partner post-service (PR = 1.26, 95% CI = 1.02, 1.55).

**Table 3:** Trauma prevalence for veterans by trauma type, life phase, and race/ethnicity

	Black (*N*=743)						Hispanic (*N*=422)						White (*N*=1919)				
	**%**	**Lower 95% CI**	**Upper 95% CI**	**Prev. Ratio**	**Lower 95% CI**	**Upper** **95% CI**	**%**	**Lower 95% CI**	**Upper 95% CI**	**Prev. Ratio**	**Lower 95% CI**	**Upper** **95% CI**	%	Lower 95% CI	Upper 95% CI	*p*	Cramer’s V
Serious Accident																	
Pre-Service	46.4^W^	42.8	50.0	0.89	0.81	0.97	51.9	47.1	56.7	0.99	0.89	1.10	52.4^B^	50.2	54.7	0.019	0.05
Service	47.0	43.4	50.6	1.03	0.94	1.12	50.9	46.2	55.7	1.11	1.00	1.24	45.8	43.5	48.0	0.152	0.04
Post-Service	44.7^HW^	41.1	48.3	1.30	1.17	1.43	37.4^B^	32.8	42.1	1.09	0.95	1.25	34.5^B^	32.4	36.6	**<0.001**	0.09
Witnessed Violent Death																	
Pre-Service	26.1^W^	23.0	29.3	1.62	1.38	1.89	22.7^W^	18.7	26.8	1.41	1.15	1.73	16.2^BH^	14.5	17.8	**<0.001**	0.11
Service	31.2	27.9	34.6	0.87	0.77	0.98	36.7	32.1	41.3	1.02	0.89	1.17	36.0	33.9	38.2	0.048	0.04
Post-Service	28.8^W^	25.5	32.1	1.27	1.10	1.46	26.3	22.1	30.5	1.16	0.97	1.39	22.7^B^	20.8	24.6	**0.003**	0.06
Community Violence																	
Pre-Service	13.7^W^	11.3	16.2	1.38	1.10	1.73	13.5	10.2	16.8	1.36	1.03	1.79	10.0^B^	8.6	11.3	**0.007**	0.06
Service	23.7	20.6	26.7	0.95	0.82	1.10	26.5	22.3	30.8	1.06	0.89	1.27	25.0	23.0	26.9	0.550	0.02
Post-Service	22.2	19.2	25.2	1.07	0.91	1.26	20.4	16.5	24.2	0.98	0.80	1.21	20.7	18.9	22.6	0.662	0.02
Captivity																	
Pre-Service	3.2	2.0	4.5	1.44	0.88	2.36	2.4	0.9	3.8	1.06	0.54	2.09	2.2	1.6	2.9	0.335	0.03
Service	2.0	1.0	3.0	1.25	0.68	2.30	2.4	0.9	3.8	1.47	0.72	2.97	1.6	1.1	2.2	0.514	0.02
Post-Service	4.8^W^	3.3	6.4	2.58	1.64	4.07	2.1	0.8	3.5	1.14	0.55	2.34	1.9^B^	1.3	2.5	**<0.001**	0.08
Sexual Assault (non-IP)																	
Pre-Service	31.5^W^	28.2	34.8	1.30	1.14	1.48	27.7	23.5	32.0	1.14	0.96	1.36	24.3^B^	22.4	26.2	**0.001**	0.07
Service	16.4	13.8	19.1	1.01	0.83	1.22	16.8	13.3	20.4	1.03	0.82	1.30	16.3	14.7	18.0	0.967	0.01
Post-Service	7.0	5.2	8.8	1.26	0.91	1.73	6.6	4.3	9.0	1.19	0.80	1.78	5.6	4.5	6.6	0.336	0.03
Physical Assault (non-IP)																	
Pre-Service	23.1	20.1	26.2	0.99	0.84	1.15	22.5	18.5	26.5	0.96	0.79	1.16	23.5	21.6	25.4	0.905	0.01
Service	16.8	14.1	19.5	0.91	0.76	1.10	15.6	12.2	19.1	0.85	0.67	1.08	18.4	16.7	20.1	0.324	0.03
Post-Service	15.6	13.0	18.2	1.24	1.01	1.53	13.7	10.5	17.0	1.09	0.84	1.43	12.6	11.1	14.0	0.115	0.04
Sexual Assault (IP)																	
Pre-Service	12.4	10.0	14.8	1.16	0.92	1.47	12.8	9.6	16.0	1.20	0.91	1.59	10.6	9.3	12.0	0.266	0.03
Service	12.0	9.6	14.3	1.00	0.79	1.26	13.3	10.0	16.5	1.11	0.84	1.45	12.0	10.5	13.4	0.754	0.01
Post-Service	14.0^W^	11.5	16.5	1.31	1.05	1.63	14.0	10.7	17.3	1.31	1.00	1.71	10.7^B^	9.3	12.1	0.023	0.05
Physical Assault (IP)																	
Pre-Service	21.5^W^	18.6	24.5	1.45	1.22	1.73	19.4	15.7	23.2	1.31	1.05	1.63	14.9^B^	13.3	16.4	**<0.001**	0.08
Service	26.5^W^	23.3	29.7	1.33	1.14	1.54	24.2	20.1	28.3	1.21	1.00	1.46	20.0^B^	18.2	21.8	**<0.001**	0.07
Post-Service	31.8^HW^	28.4	35.1	1.89	1.64	2.19	21.1^B^	17.2	25.0	1.26	1.02	1.55	16.8^B^	15.1	18.5	**<0.001**	0.15

Note. Significant pairwise comparisons use the letters ^B^, ^H^, and ^W^ to indicate a significant difference from Black, Hispanic, and White participants, respectively. These pairwise comparisons use a Bonferroni correction with a critical alpha of *p* <.0167. Prevalence ratios for Black and Hispanic groupings use White as the reference category; IP = intimate partner

### Differences in trauma exposure across ethnoracial groups for women veterans

Table 4 shows trauma exposure prevalence, prevalence ratios and 95% CIs for women Veterans by trauma type, life phase, and race/ethnicity. Black women across the full range of neighborhood contexts were more likely to experience a serious accident post-service (43.8%) than Hispanic (33.5%) or White women across the full range of neighborhood contexts (30.7%; PR = 1.43, 95% CI = 1.24, 1.65). White women were less likely to witness a violent death pre-service (11.5%) than Black (21.1%; PR = 1.83, 95% CI = 1.42, 2.37) or Hispanic (18.1%) women. These differences in witnessing violent death disappeared during service, but post-service, White women across the full range of neighborhood contexts were again less likely to witness violent death (20.0%) than Black women across the full range of neighborhood contexts (26.5%; PR = 1.32, 95% CI = 1.08, 1.62). Black women across the full range of neighborhood contexts were also more likely to report being held in captivity or kidnapped post-service (5.6%) than White women (2.0%; PR =, 2.82, 95% CI = 1.55, 5.11). During service, White women were more likely to report non-intimate partner sexual assault (30.2%) than Black women (22.7%) (PR = 0.75, 95% CI = 0.62, 0.92). Black women were more likely to report a physical assault by an intimate partner during service (34.6%) than White women (26.6%; prevalence ratio, 1.30, 95% CI = 1.10, 1.54) and also at post-service, (34.2%) than Hispanic (23.7%) or White (20.5%) women across the full range of neighborhood contexts (PR = 1.67, 95% CI = 1.39, 2.00). All other comparisons were not statistically significant.

**Table 4:** Trauma prevalence for women veterans by trauma type, life phase, and race/ethnicity

	Black (*N*=473)						Hispanic (*N*=222)						White (*N*=942)				
	**%**	**Lower 95% CI**	**Upper 95% CI**	**Prev. Ratio**	**Lower 95% CI**	**Upper 95% CI**	**%**	**Lower 95% CI**	**Upper 95% CI**	**Prev. Ratio**	**Lower 95% CI**	**Upper** **95% CI**	%	Lower 95% CI	Upper 95% CI	*p*	Cramer’s V
Serious Accident																	
Pre-Service	46.5	41.9	51.2	0.97	0.86	1.10	49.3	42.6	56.0	1.03	0.89	1.20	47.8	44.6	51.1	0.477	0.02
Service	43.8	39.2	48.4	1.15	1.01	1.32	46.5	39.8	53.2	1.22	1.04	1.45	38.0	34.8	41.2	0.023	0.07
Post-Service	43.8^WH^	39.2	48.4	1.43	1.24	1.65	33.5^B^	27.2	39.8	1.09	0.88	1.35	30.7^B^	27.7	33.7	**<0.001**	0.12
Witnessed Violent Death																	
Pre-Service	21.1^W^	17.3	24.9	1.83	1.42	2.37	18.1^W^	13.0	23.3	1.58	1.12	2.21	11.5^BH^	9.4	13.6	**<0.001**	0.12
Service	27.2	23.1	31.3	1.05	0.87	1.27	30.2	24.1	36.4	1.17	0.93	1.47	25.9	23.1	28.8	0.432	0.03
Post-Service	26.5^W^	22.4	30.6	1.32	1.08	1.62	22.3	16.8	27.9	1.11	0.84	1.48	20.0^B^	17.4	22.7	0.027	0.07
Community Violence																	
Pre-Service	11.7	8.7	14.7	1.10	0.80	1.51	13.5	8.9	18.1	1.27	0.86	1.87	10.6	8.6	12.6	0.474	0.03
Service	20.4	16.7	24.2	0.98	0.79	1.23	22.3	16.8	27.9	1.07	0.81	1.42	20.8	18.2	23.5	0.851	0.01
Post-Service	20.4	16.7	24.2	1.06	0.84	1.32	18.6	13.4	23.8	0.96	0.70	1.31	19.4	16.8	24.2	0.833	0.02
Captivity																	
Pre-Service	3.8	2.0	5.6	1.11	0.62	1.99	2.3	0.3	4.3	0.68	0.27	1.72	3.4	2.2	4.6	0.607	0.03
Service	2.0	0.7	3.3	1.07	0.48	2.39	2.3	0.3	4.3	1.24	0.46	3.31	1.9	1.0	2.8	0.914	0.01
Post-Service	5.6^W^	3.5	7.8	2.82	1.55	5.11	2.3	0.3	4.3	1.17	0.44	3.11	2.0^B^	1.1	2.9	**0.001**	0.09
Sexual Assault (non-IP)																	
Pre-Service	42.7	38.1	47.3	1.12	0.98	1.28	42.3	35.7	48.9	1.11	0.93	1.32	38.2	35.0	41.4	0.219	0.04
Service	22.7^W^	18.8	26.6	0.75	0.62	0.92	28.8	22.8	34.9	0.95	0.76	1.20	30.2^B^	27.2	33.2	**0.014**	0.07
Post-Service	8.8	6.1	11.4	0.95	0.66	1.37	8.4	4.7	12.1	0.91	0.56	1.48	9.2	7.3	11.1	0.918	0.01
Physical Assault (non-IP)																	
Pre-Service	22.5	18.6	26.4	1.21	0.97	1.51	19.5	14.2	24.8	1.05	0.78	1.42	18.6	16.1	21.1	0.244	0.04
Service	15.3	11.9	18.6	1.39	1.05	1.86	9.8	5.8	13.7	0.89	0.57	1.39	11.0	8.9	13.0	0.040	0.06
Post-Service	12.8	9.7	15.9	1.45	1.05	1.99	9.8	5.8	13.7	1.10	0.70	1.74	8.9	7.0	10.7	0.076	0.06
Sexual Assault (IP)																	
Pre-Service	17.8	14.2	21.3	0.97	0.76	1.24	20.9	15.5	26.4	1.15	0.85	1.54	18.3	15.7	20.8	0.595	0.03
Service	17.3	13.8	20.8	0.79	0.62	1.00	21.4	15.9	26.9	0.97	0.73	1.29	22.0	19.3	24.7	0.124	0.05
Post-Service	18.4	14.8	22.0	1.09	0.85	1.39	20.0	14.6	25.4	1.18	0.87	1.60	16.9	14.5	19.4	0.526	0.03
Physical Assault (IP)																	
Pre-Service	26.1	22.0	30.2	1.24	1.01	1.51	24.7	18.9	30.4	1.17	0.90	1.53	21.0	18.4	23.7	0.100	0.05
Service	34.6^W^	30.2	39.0	1.30	1.10	1.54	31.6	25.4	37.9	1.19	0.95	1.49	26.6^B^	23.7	29.5	**0.008**	0.08
Post-Service	34.2^HW^	29.7	38.6	1.67	1.39	2.00	23.7^B^	18.0	29.4	1.16	0.88	1.52	20.5^B^	17.9	23.1	**<0.001**	0.14

Note. Significant pairwise comparisons use the letters ^B^, ^H^, and ^W^ to indicate a significant difference from Black, Hispanic, and White participants, respectively. These pairwise comparisons use a Bonferroni correction with a critical alpha of *p* <.0167. Prevalence ratios for Black and Hispanic groupings use White as the reference category; IP = intimate partner

### Differences in trauma exposure across ethnoracial groups for men veterans

Table 5 shows trauma exposure prevalence, prevalence ratios and 95% CIs for men Veterans by trauma type, life phase, and race/ethnicity. White men were more likely to report being involved in a serious accident pre-service (56.4%) than Black men (46.1%; PR = 0.82, 95% CI = 0.71, 0.94). Pre-service, White men were less likely to witness violent death (20.1%) than Black men (33.5%; PR = 1.66, 95% CI = 1.35, 2.04). Black men were more likely to report exposure to community violence pre-service (16.9%) than White men (9.2%; PR = 1.84, 95% CI = 1.33, 2.54). With respect to experiencing intimate partner physical assault, Black men had higher prevalence estimates pre-service (14.8%) and post-service (27.1%) than White men (9.0% and 13.8%; PRs = 1.64, 95% CI = 1.17, 2.32 and 1.96, 95% CI = 1.53, 2.51). All other comparisons were not statistically significant.

**Table 5:** Trauma prevalence for men veterans by trauma type, life phase, and race/ethnicity

	Black (*N*=291)						Hispanic (*N*=210)						White (*N*=994)				
	**%**	**Lower 95% CI**	**Upper 95% CI**	**Prev. Ratio**	**Lower 95% CI**	**Upper** **95% CI**	**%**	**Lower 95% CI**	**Upper 95% CI**	**Prev. Ratio**	**Lower 95% CI**	**Upper** **95% CI**	%	Lower 95% CI	Upper 95% CI	*p*	Cramer’s V
Serious Accident																	
Pre-Service	46.1^W^	40.3	51.9	0.82	0.71	0.94	55.0	48.1	61.8	0.97	0.85	1.12	56.4^B^	53.3	59.6	**0.009**	0.08
Service	51.1	45.2	56.9	0.96	0.85	1.10	56.4	49.6	63.3	1.07	0.93	1.22	53.0	49.8	56.1	0.500	0.03
Post-Service	45.4	39.6	51.2	1.18	1.02	1.37	41.6	34.8	48.4	1.08	0.90	1.30	38.4	35.4	41.5	0.099	0.06
Witnessed Violent Death																	
Pre-Service	33.5^W^	28.0	38.9	1.66	1.35	2.04	27.7	21.5	33.9	1.38	1.07	1.78	20.1^B^	17.6	22.7	**<0.001**	0.13
Service	37.3	31.7	43.0	0.82	0.70	0.97	44.1	37.2	50.9	0.97	0.82	1.15	45.4	42.3	48.5	0.054	0.06
Post-Service	31.7	26.3	37.1	1.25	1.03	1.54	30.2	23.9	36.5	1.20	0.94	1.51	25.3	22.5	28.0	0.059	0.06
Community Violence																	
Pre-Service	16.9^W^	12.5	21.3	1.84	1.33	2.54	13.9	9.1	18.6	1.51	1.01	2.24	9.2^B^	7.4	11.0	**<0.001**	0.10
Service	29.2	23.9	34.5	1.01	0.82	1.24	31.7	25.3	38.1	1.09	0.87	1.37	29.0	26.2	31.9	0.751	0.02
Post-Service	24.6	19.6	29.7	1.12	0.89	1.42	21.8	16.1	27.5	0.99	0.74	1.32	22.0	19.4	24.6	0.619	0.03
Captivity																	
Pre-Service	2.1	0.4	3.8	1.72	0.65	4.55	2.5	0.3	4.6	2.02	0.72	5.66	1.2	0.5	1.9	0.308	0.04
Service	1.8	0.2	3.3	1.43	0.51	4.04	2.5	0.3	4.6	2.02	0.72	5.66	1.2	0.5	1.9	0.383	0.04
Post-Service	3.5	1.4	5.7	2.03	0.94	4.37	2.0	0.1	3.9	1.14	0.39	3.35	1.7	0.9	2.6	0.183	0.05
Sexual Assault (non-IP)																	
Pre-Service	13.7	9.7	17.7	1.17	0.83	1.64	12.9	8.2	17.5	1.09	0.74	1.63	11.8	9.7	13.8	0.648	0.02
Service	5.3	2.7	7.9	1.57	0.86	2.84	3.5	0.9	6.0	1.03	0.46	2.29	3.4	2.2	4.5	0.322	0.04
Post-Service	3.9	1.6	6.1	1.72	0.85	3.51	5.0	2.0	7.9	2.20	1.06	4.58	2.2	1.3	3.2	0.068	0.06
Physical Assault (non-IP)																	
Pre-Service	24.3	19.3	29.3	0.87	0.69	1.09	25.7	19.7	31.8	0.92	0.71	1.19	27.9	25.1	30.7	0.443	0.03
Service	18.7	14.1	23.2	0.74	0.56	0.96	21.8	16.1	27.5	0.86	0.65	1.14	25.4	22.6	28.1	0.053	0.06
Post-Service	20.1	15.4	24.7	1.23	0.94	1.62	17.8	12.5	23.1	1.10	0.79	1.52	16.3	13.9	18.6	0.316	0.04
Sexual Assault (IP)																	
Pre-Service	4.2	1.9	6.6	1.33	0.69	2.56	4.0	1.3	6.7	1.25	0.58	2.68	3.2	2.1	4.3	0.642	0.02
Service	3.5	1.4	5.7	1.32	0.65	2.71	5.0	2.0	7.9	1.86	0.91	3.80	2.7	1.6	3.7	0.217	0.05
Post-Service	6.7	3.8	9.6	1.34	0.80	2.23	7.9	4.2	11.6	1.58	0.92	2.72	5.0	3.6	6.4	0.201	0.05
Physical Assault (IP)																	
Pre-Service	14.8^W^	10.7	18.9	1.64	1.17	2.32	12.9	8.2	17.5	1.43	0.95	2.16	9.0^B^	7.2	10.8	**0.011**	0.08
Service	14.1	10.0	18.1	1.01	0.73	1.40	15.3	10.4	20.3	1.10	0.77	1.58	13.9	11.7	16.1	0.866	0.01
Post-Service	27.1^W^	21.9	32.3	1.96	1.53	2.51	18.3	13.0	23.7	1.33	0.95	1.85	13.8^B^	11.6	16.0	**<0.001**	0.14

Note. Significant pairwise comparisons use the letters ^B^, ^H^, and ^W^ to indicate a significant difference from Black, Hispanic, and White participants, respectively. These pairwise comparisons use a Bonferroni correction with a critical alpha of *p* <.0167. Prevalence ratios for Black and Hispanic groupings use White as the reference category. IP = intimate partner

## Discussion

This study provides novel information about the prevalence of Veterans’ trauma exposure within three distinct life phases (e.g., pre-service, during service, post-service) and across varying, intersectional demographic subgroups (e.g., gender, race/ethnicity) from August 2018 to March 2022. Findings are derived from a unique national, longitudinal study that oversampled women Veterans and Veterans living in high crime communities in order to capture those members of subgroups of the Veteran population that are most vulnerable to experiencing trauma, particularly interpersonal trauma.

### Gender

In comparing subgroups of the study sample, the most differences by far emerged between women and men. The findings are largely consistent with the extant literature. For example, in line with epidemiological study data, men were more likely to experience a serious accident, physical assault by someone other than an intimate partner, and witness violent death across all three life phases [3, 6]. Women, on the other hand, were more likely to experience physical and sexual assault by an intimate partner, and sexual assault by someone other than an intimate partner across all three life phases [3, 6]. Not surprisingly, prevalence ratios were especially high for women’s exposure to sexual assault perpetrated by both an intimate partner and someone other than an intimate partner across all life phases. Notably, reported exposure to sexual assault perpetrated by any assailant during service was over 6 times greater for women than men. Women’s elevated prevalence of sexual assault during service is consistent with extant research on MST, indicating that 24% of women versus 2% of men experience MST characterized by sexual assault [22]. It is important to note that reports of MST have reliably been considered underestimates, particularly amongst men, and may be higher than indicated in this study [22]. Overall, findings highlight that observed gender differences for various types of trauma exposures remain consistent across the lifespan in this Veteran sample and are in line with previous research.

For two types of traumatic events, however, gender differences emerged only during certain life phases. Prior to entering service, women Veterans were more likely than men Veterans to be held captive, although captivity was a rare event across all life phases for both genders (3.5% vs. 2.0%). When examining trauma exposure during service, men were more likely than women to experience community violence. This differential, elevated prevalence of community violence (e.g., terrorist attacks, bombings, riots) for men during military service may be due to their roles in the military. As barriers to women’s assignments to combat units and occupations have recently been removed with the repeal of the Ground Combat Exclusion Assignment Rule, gender differences in exposures to these types of traumas may diminish with time [23].

In summary, in this study, men and women Veterans differed in prevalence of exposure across every type of trauma and these gender differences were observed consistently across nearly every life phase. The consistency of the patterns of exposures across lifespan suggests that gender is an important factor in trauma exposure, irrespective of life phase with few exceptions.

### Race/Ethnicity

Differences between ethnic and racial groups clearly emerged across trauma types in this study. Notably, within trauma types, Black and (to a lesser extent) Hispanic Veterans were more likely to experience most trauma types compared to White Veterans, with the single exception of experiencing a serious accident. Compared to the extent of gender differences that emerged consistently across life phases, fewer differences emerged between ethnoracial groups. When subgroups differed, differences were most often observed prior to and/or after service. The only trauma type that showed consistent prevalence patterns across all three life phases was the experience of physical assault by an intimate partner with Black Veterans more likely to experience this trauma than White Veterans. Prior to entering service, Black Veterans were more likely than White Veterans to experience community violence and experience sexual assault from someone other than an intimate partner, but those group differences disappeared during and after service. Different patterns emerged when examining trauma exposure post-service; Black Veterans across the full range of neighborhood contexts were more likely than White Veterans across the full range of neighborhood contexts to experience a serious accident and be held captive. Black Veterans were more likely to witness violent death before and after service but not during service. In fact, the only difference to emerge during service was a higher prevalence of physical assault by an intimate partner for Black Veterans compared to White Veterans.

In summary, in this study, when differences in trauma exposure across race and ethnicity were examined, groups differed by trauma type, but those differences were not consistent across life phases. Group differences in trauma exposure during pre-service and post-service occurred most frequently but differed by trauma. There were virtually no ethnoracial differences in reported exposure to any type of trauma during service (except physical assault by an intimate partner). Exposure to physical assault by a non-intimate partner was the only trauma type where no ethnoracial differences emerged across all life phases.

### Gender and Race/Ethnicity

By applying an intersectional lens, nuanced differences in trauma exposure across race/ethnicity and gender emerged in this study, yielding important information about differential prevalence estimates for subgroups of Veterans within each life phase. As described, clearly women Veterans were consistently more likely to experience IPV and sexual assaults across the lifespan compared to men Veterans. Few ethnoracial differences emerged among the women Veterans on these types of traumas with three exceptions. Specifically, two group differences emerged during service (White women more likely to experience non-IPV sexual trauma compared to Hispanic and Black women; Black women more likely to experience IPV physical assault compared to White women) and one difference emerged after service (Black women and Hispanic women across the full range of neighborhood contexts were more likely to experience physical assault by an intimate partner). Indeed, prevalence estimates in trauma exposure prior to service was remarkably similar across all three ethnoracial groups in our women Veteran sample, with the exception of witnessing violent deaths. While all three subgroups of women Veterans were more likely to witness violent death during service, both Black and Hispanic women Veterans were more likely to experience this trauma prior to service.

The men Veterans in this study also reported more similarities than differences in exposures to different types of traumas across ethnoracial subgroups, with some important exceptions. Interestingly, there were virtually no ethnoracial differences in reported exposure to any type of trauma among subgroups of men during service. In the post-service phase, men across the full range of neighborhood contexts only differed in their experiences of exposure to physical assault by an intimate partner such that Black men had higher prevalence estimates compared to White men. Four out of the five differences that emerged among the subgroups of men occurred prior to service. Black men reported higher prevalence of exposure to community violence, witnessing violent death, and experiencing physical assault by an intimate partner prior to service compared to White men, while White men reported higher prevalence of serious accidents prior to service as compared to Black men.

These intersectional findings reinforce the notion that a person’s identit(ies) and social environment may contribute to differential trauma exposure prevalence thereby increasing the likelihood for related development of mental health problems [24]. Incorporating this lens into clinical care may inform treatment decisions [25].

Although this study sheds light on which subgroups of Veterans are more likely to experience different types of traumas at different life phases, it cannot explain the reasons for these differences. There are a host of additional factors that might also contribute to and explain these observed differential prevalence estimates. For example, epidemiological studies suggest there are a number of sociodemographic factors associated with trauma exposure, including not being married, older age, having received less education, and prior trauma exposure [6]. Minoritized populations may be more likely to experience some of these risk factors that contribute to differences in trauma exposure, given the negative impact of historical oppression on minoritized individuals [26]. Historical, structural, and institutional influences contribute to oppressive environments (i.e., high crime, environmental injustice, increased interpersonal oppression) [24, 27] that increase the likelihood of exposure to trauma inequitably for minoritized populations. Further research might seek to begin to explore the nuanced reasons for differences in trauma exposures in this Veteran sample.

Understanding gender, racial, and ethnic differences in exposure to traumatic events is complex. Historical, structural, institutional, and sociopolitical influences contribute to the larger context in which traumas occur and are experienced. These influences are not experienced equally across subgroups of the population and likely contribute to the differences in trauma exposure observed in this study. Assessing experiences of traumas, as defined by the Diagnostic and Statistical Manual of Mental Disorder (5th ed.; DSM–5 TR) [31] in this study, identified clear subgroup differences at key life phases in the Veteran population. These findings suggest that further study should seek to identify and assess the prevalence of expanded definitions of traumatic events that include culturally relevant stressors such as racism and discrimination. The cumulative effect of exposure to these gender and race-based types of experiences, not only as independent traumas, but also as critical contextual factors that have the potential to amplify and fundamentally modify Criterion A events as currently defined, warrants additional research.

Taken together, findings support previous studies suggesting that Veterans are likely to experience trauma across life phases. This overall elevated prevalence (and differential prevalence amongst subgroups) might be explained in a number of ways. First, men who come from disadvantaged circumstances (e.g., lower socioeconomic status, less education, nontraditional family structure), have low social support, and report a history of adolescent fighting, are more likely to enlist in the U.S. volunteer military [31]. Military service may also offer an escape for those living in volatile, impoverished, abusive, and/or under-privileged environments. This may be particularly true for women [28] and may explain the observed, elevated prevalence of interpersonal trauma exposure prior to service. Service within the military is, by definition, a job that incurs a higher probability of trauma exposure than many other professions. These workplace conditions and associated tasks and duties clearly increase the likelihood of trauma exposure, and prevalence estimates may differ across groups, depending on occupation within the military and certain historic limitations (such as those placed on the roles of women in the military) to participating in those occupations. The elevated prevalence of sexual trauma during military service is well-established, particularly for women, and certainly the unique military environment contributes to likelihood of exposure [29]. Finally, the high prevalence of reported exposure to trauma post-service in this study must be considered within the context of the sampling framework (i.e., oversampled for women Veterans and Veterans living in high crime communities) and timeframe. Data collection for this study took place during the COVID-19 pandemic, which has been associated with increases in various types of violence (e.g., increase in homicide rates [35], hate crimes targeting Black people [36], and domestic violence [37]) and, therefore, may have increased the likelihood of trauma exposure for women, Black and Hispanic Veterans, and Veterans living in high crime areas. Additionally, the nature of this sample may accentuate and/or mask critical information related to the prevalence of trauma exposure among Veterans. For example, our oversampling of Veterans in high crime communities may also have implications for our findings, particularly regarding reported exposure to community violence. Such considerations may help provide some context as to why subgroup differences emerged in this study, though, additional research is needed for further clarification.

### Limitations and future directions

This study is not without limitations. Despite our exploration of the intersection of gender and racial/ethnic groups, study analyses were limited to two genders (cisgender men and women) and three races/ethnicities (Black, Hispanic, White) due to insufficient sample sizes of other groups. This suggests that our examined intersectional groups may not fully represent the experiences of Veterans with additional identities that can impact social position (e.g., lesbian, gay, bisexual, transgender, queer, and other identities) (18,30). We also did not focus on other demographic and related variables (e.g., income, branch of military service) that likely contribute to and perpetuate differences in trauma exposure among subgroups. Future studies should include causal research that attempts to disentangle these effects. Moreover, our analyses only used White individuals as the referent group in our comparisons, and we did not compare men to women within different races/ethnicities (e.g., Black women vs. Black men) given sample size limitations and the large number of comparisons conducted and concern for false positive results. Future research might extend these findings by examining additional intersectional variables or samples with more diverse groups that allow for additional comparisons. Additionally, though it is a strength that our oversampling methods allowed us to make gender comparisons and examine trauma exposure among Veterans from a range of neighborhood contexts, our sample is not nationally representative and results may have limited generalizability. Our observed prevalence estimates may be overestimates due to oversampling veterans who live in high crime areas. However, the consistency of our post-service prevalence estimates (when participants are confirmed to have lived in high crime areas) with the estimates from other life phases when participants were less likely to live in a high crime area (e.g., during service) somewhat assuages this concern. Further, it is a strength that this study assessed for community violence exposure. Yet, it is possible that our community violence item and other LEC-5 items may not have captured all possible forms of trauma exposure across all trauma types. It is also possible that certain trauma exposures could fall within multiple trauma categories due to the LEC-5’s limited assessment of the context in which trauma exposures occurred (e.g., witnessing violent death could also be classified as community violence depending on context). Therefore, researchers in future studies should consider continuing to conduct the most nuanced examination of trauma exposure among Veterans that their methodology allows. Finally, since our study data were collected via survey, study findings may be subject to Veterans’ response biases and error in retrospective recall. Nonetheless, this study relied on well validated self-report instruments.

## Conclusion

This study examined gender and ethnoracial differences in Veterans’ trauma exposure across distinct life phases and using an intersectional lens. Findings revealed significant differences in trauma exposure in our Veterans sample depending on Veterans’ intersectional gender and ethnoracial identity as well as life phase. They highlight the need for targeted intervention and treatment programs to address the needs of different Veteran subgroups across life phases. Importantly, they further corroborate previous findings demonstrating elevated prevalence of assault among women Veterans and various forms of trauma exposure among Black Veterans across life phases [12, 14, 16]. These findings contribute to ongoing efforts to increase equitable access to IPV and trauma screening as well as effective trauma treatment for minoritized Veterans [30–33]. They demonstrate the critical need in ensuring that trauma-focused prevention and treatment services are culturally-sensitive and responsive to diverse Veterans and that policies support access to mental health services for such Veterans. Further, findings underscore the importance of intersectionality in data analysis and have significant implications for the conclusions that can be drawn from research on Veterans’ trauma exposure by highlighting the critical association between one’s identities and experiences. Research that ignores Veterans’ multiple identities may fail to truly understand Veterans’ traumatic experiences.

## Data Availability

Although not publicly available due to privacy restrictions, data can be made available upon reasonable request and execution of a data use agreement between sponsoring institutions.

## Abbreviations

CI: Confidence interval
IPV: Intimate partner violence
LEC-5: Life Events Checklist-5
LIGHT: Longitudinal Investigation of Gender, Health, and Trauma
MST: Military sexual trauma
PR: Prevalence ratio
VA: Department of Veterans Affairs
VADIR: Veterans Affairs/Department of Defense Identity Repository
